# Geochemical Evidence for the Control of Fire by Middle Palaeolithic Hominins

**DOI:** 10.1038/s41598-019-51433-0

**Published:** 2019-10-25

**Authors:** Alex Brittingham, Michael T. Hren, Gideon Hartman, Keith N. Wilkinson, Carolina Mallol, Boris Gasparyan, Daniel S. Adler

**Affiliations:** 1 Department of Anthropology, University of Connecticut, Storrs, CT USA; 2 Department of Geoscience, University of Connecticut, Storrs, CT USA; 3 Department of Chemistry, University of Connecticut, Storrs, CT USA; 4 Center for Environmental Science and Engineering, University of Connecticut, Storrs, CT USA; 5 Department of Archaeology, Anthropology and Geography, University of Winchester, Winchester, SO22 4NR United Kingdom; 6 Palaeolithic Hunter-Gatherer Societies Research Group, Universidad de La Laguna, Tenerife, Spain; 7 U.D.I. de Prehistoria, Arqueología e Hª Antigua (Dpto. Geografía e Historia), Universidad de La Laguna, Tenerife, Spain; 8 Archaeological Micromorphology and Biomarkers (AMBI Lab), Instituto Universitario de Bio-Orgánica Antonio González, Universidad de La Laguna, Tenerife, Spain; 9 Institute of Archaeology and Ethnography, National Academy of Sciences of the Republic of Armenia, Charents 15, Yerevan, Armenia

**Keywords:** Palaeoecology, Archaeology

## Abstract

The use of fire played an important role in the social and technological development of the genus *Homo*. Most archaeologists agree that this was a multi-stage process, beginning with the exploitation of natural fires and ending with the ability to create fire from scratch. Some have argued that in the Middle Palaeolithic (MP) hominin fire use was limited by the availability of fire in the landscape. Here, we present a record of the abundance of polycyclic aromatic hydrocarbons (PAHs), organic compounds that are produced during the combustion of organic material, from Lusakert Cave, a MP site in Armenia. We find no correlation between the abundance of light PAHs (3–4 rings), which are a major component of wildfire PAH emissions and are shown to disperse widely during fire events, and heavy PAHs (5–6 rings), which are a major component of particulate emissions of burned wood. Instead, we find heavy PAHs correlate with MP artifact density at the site. Given that hPAH abundance correlates with occupation intensity rather than lPAH abundance, we argue that MP hominins were able to control fire and utilize it regardless of the variability of fires in the environment. Together with other studies on MP fire use, these results suggest that the ability of hominins to manipulate fire independent of exploitation of wildfires was spatially variable in the MP and may have developed multiple times in the genus *Homo*.

## Introduction

The use of fire played a key role in the evolution of the genus *Homo*^1^, allowing for warmth, cooking, birch tar production, protection from predators, a venue for social interactions and access to high latitudes and dark caves^2^. Evidence of hominin fire use is present in the archaeological record beginning around 1.5 million years ago^3–5^, and while it has long been assumed that a variety of hominin species could use fire^5–7^, the degree to which hominins were able to intentionally create and control fire (pyrotechnology) is debated^8–17^. Recently, some have argued that this ability was exclusive to modern humans^9,12,16^, with other hominins, such as Neanderthals, limited to exploiting wildfires. Neanderthals went extinct during the late Pleistocene. The reasons for that extinction remain unclear, and recent genetic data indicate that Neanderthal DNA persists among certain modern populations of *Homo sapiens*^18^. Neanderthal extinction has been linked specifically or in combination to fire use, foraging behaviors, a lack of clothing, demography, climate change, and interactions with expanding populations of Upper Palaeolithic *Homo sapiens*^19,20^. Evidence for the use of fire among MP hominins includes burning found in archaeological sites, and the construction of hearths. Manganese dioxide blocks, which are useful for fire-starting, have also been excavated at MP sites and are interpreted by some as evidence of Neanderthal fire production^21^. However, research at MP sites in France claims that fire frequency, measured by thermally altered flint and burnt bone, is positively correlated with warmer periods, when wildfire frequency is assumed to be highest, rather than with colder, glacial periods when fire use would have provided greater benefit; this correlation is derived by associating chronometrically dated stratigraphic layers at these sites with particular phases of Pleistocene global temperature records. These data are collectively interpreted as evidence that Neanderthals had not mastered pyrotechnology, and instead harvested natural fires caused by lightning strikes^12,16^, though this interpretation is not accepted by all^15^.

In order to test the hypothesis that MP hominin fire use was correlated with natural fire frequency, we developed a record of polycyclic aromatic hydrocarbons (PAHs) for the MP site of Lusakert Cave 1 (LKT1) in the Armenian Highlands from eighteen sedimentary units associated with MP lithic technology^22–24^. Organic molecular markers of fire, including PAHs, can provide a quantitative record of fire over geological time scales^25,26^, though lighter PAHs may be more susceptible to the effects of degradation due to their higher solubility^27^. During biomass combustion PAHs with variable structure are formed typically containing two (e.g. naphthalene) to six (e.g. benzo[*g, h, i*]perylene) rings (Supplementary Fig. S1). A number of studies have documented the PAH emission from wood burning in hearths and fireplaces^28–33^. While varied PAHs are produced during wood combustion, low molecular weight PAHs (lPAHs) with four or fewer rings concentrate in the gaseous phase, whereas high molecular weight PAHs (hPAHs), with five or more rings tend to concentrate in the particulate phase^34–36^. Hearths in archaeological contexts can reach maximum temperatures of 1000 °C with mean temperatures of 600 °C, which provides sufficient energy for the production of hPAHs^37,38^.

Unlike wood combustion in hearths, studies document the low production of hPAHs in wild fires of varying intensities, which produce mostly 3- and 4-ring PAHs. For example, soils measured in a recently burned area in southern France did not measure hPAH abundances higher than control samples^39^. Following forest fires in South Korea, lPAH concentrations were found to be 3 to 28 times higher than hPAH concentrations^40^. Soil O-horizons in Russia analyzed two, ten and 16 years after a wildfire found that hPAH concentrations were not higher than background soils, despite 9-fold increases in total PAH concentrations^41^. hPAHs comprised 35% of the total PAH load in unburnt samples from fire prone regions in Spain, but less than 10% in burnt soils after wildfires due to the addition of lPAHs^42^. Finally, in savannah fires in Australia the frequency of emitted lPAHs was 13 to 30 times greater than hPAHs^43^. These data attest to the widespread production and dispersal of lPAHs associated with wildfire events.

In sedimentary records from fire-prone regions, hPAHs are typically lower in abundance than lPAHs^44,45^. Different explanations have been given for the lack of hPAHs relative to lPAHs, including changes in temperature or burn intensity^44^, as higher molecular weight PAHs require higher activation energies for synthesis. Another potential explanation is the proximity to the source of the fire^45^, as hPAHs are less likely to travel from the source of the fire. Therefore, in the context of a spatially confined archaeological site such as LKT1, we expect that the primary mode of deposition of lPAHs will be through long-range dispersals of wildfire, whereas the primary mode of deposition of hPAHs will be local fire use within the cave by hominins. Given the low abundance of hPAHs produced during wildfires relative to lPAHs, and their concentration in the particulate emissions from wood burned in hearths, it is the most parsimonious explanation that accumulation of hPAHs in the sediments of archaeological sites like Lusakert Cave will be due to increased particulate deposition of residues from wood combustion. These particulate emissions of hearths will concentrate locally, on the scale of 10 s of meters.

**Figure 1:**
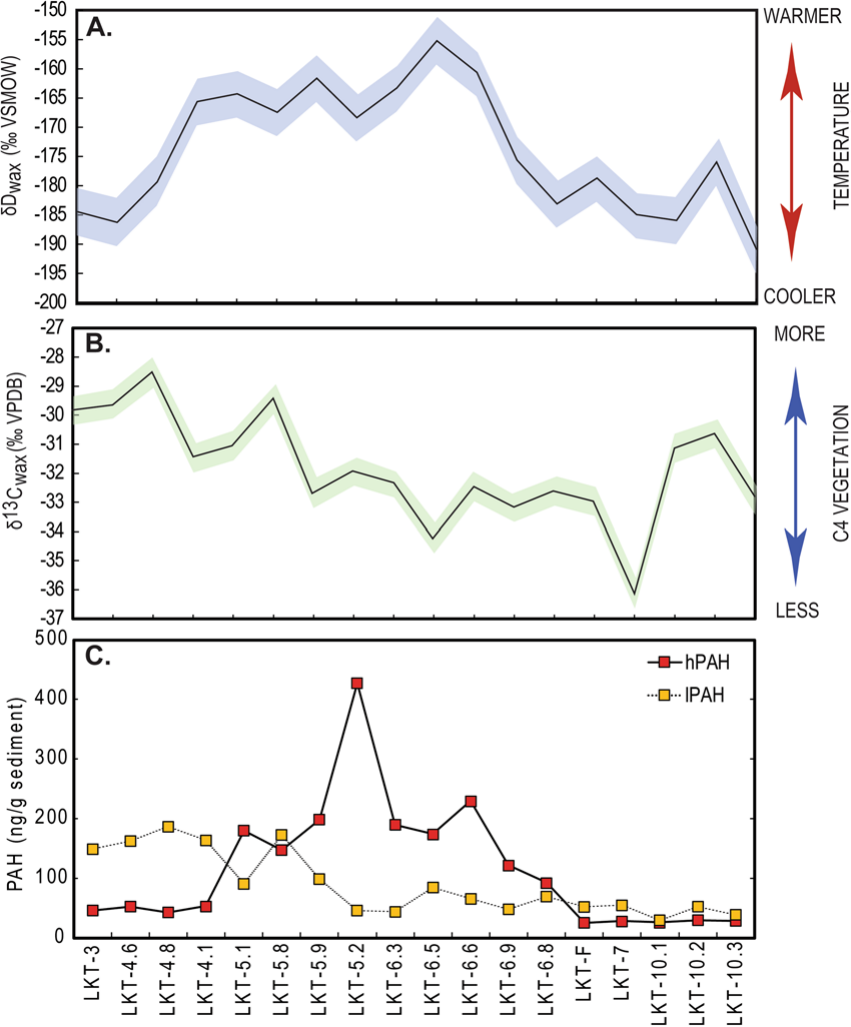
(**A**) δD_wax_, (**B)** δ^13^C_wax_, (**C**) concentrations of hPAHs (red squares), and lPAHs (orange squares) from each sedimentary Unit at LKT1. δD_wax_ (4‰) and δ^13^C_wax_ (0.5‰) values are plotted with 1σ error bars. LKT1 stratigraphic layers are oriented oldest (right) to youngest (left).

In addition to PAH data for local or regional fire, we also analyzed the hydrogen (δD_wax_) and carbon (δ^13^C_wax_) isotope composition of long-chain *n*-alkanes, the molecular remains of epicuticular waxes of terrestrial plants, to constrain regional climate and hydrology through the period of hominin occupation (Fig. 1A,B). Like hPAHs and lPAHs, *n*-alkanes are preserved over geological time scales. Long-chain *n*-alkanes at LKT1 have high odd-over-even predominance (OEP), demonstrating that they did not undergo significant microbial^46^ or thermal^47^ alteration (Supplementary Table S2). The δ^13^C values in plant material is primarily a reflection of the photosynthetic pathway of the plant (C_3_, C_4_ or CAM). In C_3_ plants, δ^13^C values are influenced by physiological changes as plants balance water loss and CO_2_ uptake through the regulation of stomata, altering the partial pressures of intracellular (C_*i*_) relative to extracellular (C_*a*_) CO_2_^48,49^, causing a positive shift in δ^13^C values in C_3_ plants experiencing water stress^50^. Fractionation during lipid biosynthesis causes *n*-alkane δ^13^C values to be lower relative to bulk plant tissues^51,52^. Therefore, sedimentary δ^13^C_wax_ values of *n*-alkanes predominantly reflect plant photosynthetic type and factors that affect isotopic discrimination during carbon fixation^53^. δD_wax_ record the isotope values of ambient water during the period of growth^54^, reflecting mostly precipitation isotope values in terrestrial systems. δD values of precipitation are influenced by temperature, amount of precipitation, and cloud transport history^55,56^. Most of the variability in modern precipitation δD values in the Armenian Highlands is explained by changes in temperature, and there is no significant trend associated with amount of precipitation^57^. This is also documented in Global Network of Isotopes in Precipitation stations in Georgia and Turkey (Supplementary Fig. S3). Hydrogen undergoes further fractionation during lipid biosynthesis, which is influenced by the timing of wax formation, plant physiology and functional type^58–60^.

LKT1 was excavated by an Armenian-American-British team between 2008 and 2011 and is one of the few sites in the region that preserves stratified assemblages of lithic and faunal material (Supplementary Discussion S4, Supplementary Fig. S5). In addition, there is extensive evidence for fire in the form of charcoal and visible combustion structures, as well as thermally altered bone micro-fragments in micromorphological samples (Supplementary Figs S6, S7, S8). The lithic assemblage is made entirely of obsidian from a variety of local (<25 km) and exotic (>25 km) sources, it is based on the Levallois method, and it can be classified as Middle Palaeolithic, a toolkit traditionally associated with Neanderthals in the Caucasus and neighboring regions^22,23^. Preliminary luminescence and AMS radiocarbon dates, as well as tephrochronological correlations constrain the stratigraphy of the site to 60–40 ka.

## Results and Discussion

At LKT1, δ^13^C_wax_ values increased during the period of deposition (Fig. 1A), which is best explained by an increase in the proportion of C_4_ vegetation or C_3_ vegetation undergoing water stress. δD_wax_ shows a clear shift to more positive values in the middle of the depositional sequence (Fig. 1B), which modern data show is best explained by changes in regional or global temperature through the period of deposition. By coupling these palaeoenvironmental proxy data with molecular records of local and regional fire intensity, we can test whether fire use among MP hominins was predicated on climate (natural availability) or behavior (pyrotechnology). If climate, as recorded by δD_wax_ and δ^13^C_wax_, dictated fire use by hominins, we would expect a correlation between natural fire availability (lPAHs) and on-site fire frequency (hPAHs). If hominin pyrotechnology drove fire use, we would not expect to see a strict correlation between climate and fire frequency.

**Figure 2:**
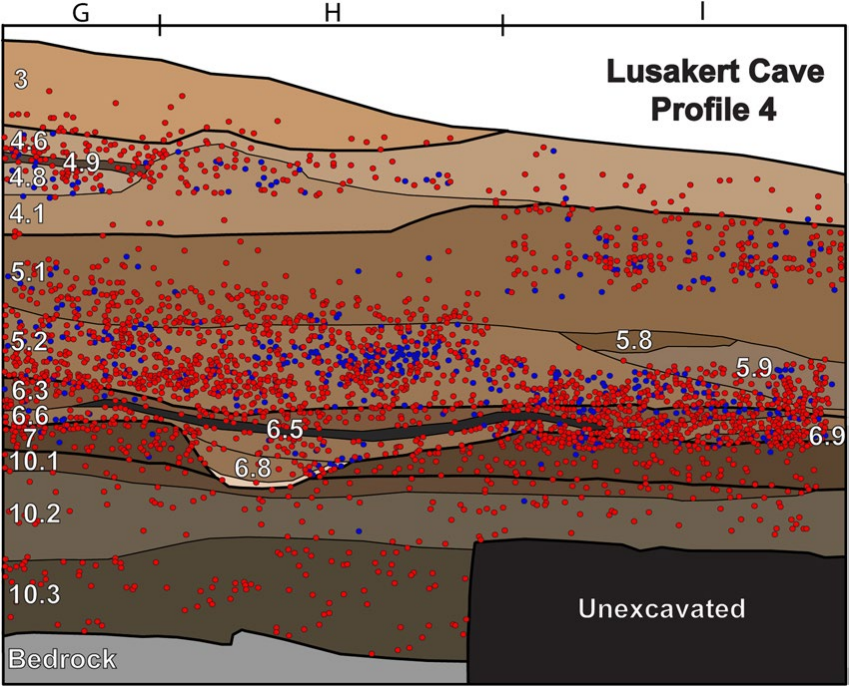
Distribution of measured lithic (red circles) and faunal remains (blue circles) excavated by stratigraphic unit from LKT1 during the 2008–2011 field seasons. 1 meter excavation squares (G, H and I) are labeled. Sedimentary units are labeled in white. Please reference Extended Data Figs 1, 2 for profile location.

**Figure 3:**
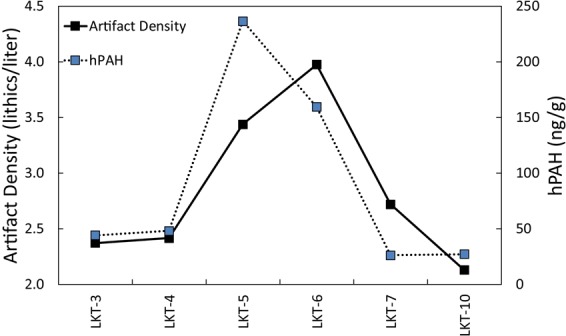
Artifact density of plotted lithic artifacts and hPAH concentrations of each layer at LKT1. Average hPAH concentrations of subunits are nested into their unit designations.

The combined record of local fire (hPAHs), regional fire (lPAHs), vegetation (δ^13^C_wax_) and temperature (δD_wax_) change documented at LKT1 demonstrates that MP hominins used fire in the cave during periods of low wildfire abundance, as recorded by lPAHs (Fig. 1). We find that there is no significant correlation (Spearman’s ρ = 0.141, p = 0.575) between lPAHs and hPAHs within samples, which decouples local fire frequency at the site from regional fire frequency (Fig. 1C, Supplementary Table S9). Layers 5 and 6, where the highest concentrations of hPAHs were measured, correspond with low frequencies of lPAHs. High concentrations of hPAHs were not always measured in archaeological layers with visible hearth features or other indications of fire use, for example in Layer 4 which contains abundant charcoal fragments and burnt bone fragments. In addition, high concentrations of hPAHs in Layers 5 and 6 correspond to the highest artifact densities, which can be correlated with increased occupation intensity (Fig. 2). The inverse is documented in strata above and below Layers 5 and 6 (i.e. Layers 3, and 10.1–10.3), where artifact density and hPAHs are lowest. This relationship between PAHs and artifact concentration is true no matter how we normalize the PAH concentration (to sediment weight or *n*-alkane concentration) and is not dependent on sediment type (Supplementary Fig. S10). These data show that since artifact density is correlated with hPAH concentrations (Fig. 3, Pearson’s *r* = 0.826, p < 0.05) rather than wild fire frequency, MP hominins at the site were able to habitually control fire, and that these activities were independent of climate (i.e. natural fires).

At LKT1, the most abundant hPAHs are B(a)P (8 layers), B(k)F (8 layers) and B(b)F (2 layers). In a series of experiments on wood combustion in fireplaces and wood stoves, B(a)P is the most abundant hPAH produced of those measured in this study in 20 of the 29 different species of wood with B(k)F the most abundant in 8 species and B(b)F the most abundant in 1^29,61–63^. Overall, the abundance of hPAHs in these modern wood combustion studies is (in order most abundant to least): B(a)P > B(k)F > B(b)F > I(1,2,3-cd)P + D(a, h)A > B(g, h, i)P. This order of abundance is the same as the averaged abundance for the layers at Lusakert, B(a)P > B(k)F > B(b)F > I(1,2,3-cd)P + D(a, h)A > B(g, h, i)P (Supplementary Fig. S11). The relationship between the abundances of different hPAHs at LKT1 and those emitted from burnt wood is significant (Pearson’s *r* = 0.861, p < 0.05). B(a)P does seem to be more abundant relative to other hPAHs than in the modern wood burning study, however, other studies demonstrate similar high abundances of B(a)P relative to other hPAHs in burning of pine wood with green needles and savanna grasses (Supplementary Fig. S11)^64^. lPAH abundance at LKT1 is similar to those of gaseous emissions of open burns of straw (Pearson’s *r* = 0.742, p < 0.10), which may be a reflection of the open environment suggested by the high δ^13^C_wax_ values^65^.

Other potential causes for the inversion of the relative concentration of hPAH and lPAH abundance in Units 5 and 6 at LKT1 include the loss of lPAHs due to postdepositional effects. The solubility of hydrocarbons like PAHs and *n*-alkanes are correlated with their molecular weight, and as such lower molecular weight PAHs and *n*-alkanes are more susceptible to degradation. Denis *et al*.^27^ argue that sedimentary units with lPAH concentrations greater than hPAH concentrations are indicative of favorable conditions for organic preservation^27^. This is observed in Units 3, 4, 7 and 10 at Lusakert, but not in Units 5 and 6. However, based on the consistently high OEP values, *n*-alkanes at the site do not show any increase in degradation in Units 5 and 6 (Supplemental Information 2). We do not expect changes in preservation potential based on lithologies of the sedimentary units given the relatively consistent depositional environment and grain size through the sequence (Supplemental Information 4). Given the preservation of other hydrocarbons, we argue that the changes in hPAH concentrations at LKT1 is reflective of changes in PAH production, rather than variable preservation.

This coupled record of fire and climate also shows the drivers of fire frequency in the past in the Armenian Highlands. There is a significant positive correlation between lPAHs and δ^13^C_wax_ values (Spearman’s ρ = 0.513, p = 0.029) and no significant relationship with δD_wax_ values (Spearman’s ρ = 0.210, p = 0.403), suggest wild fire frequency (lPAHs) is not determined by temperature, but rather by changes in vegetation. More positive δ^13^C_wax_ values, representing more open habitats, are associated with higher frequencies of wild fire, whereas more negative δ^13^C_wax_ values, associated with more closed habitats, are associated with lower frequencies of wildfire. Other studies have observed this correlation between δ^13^C_wax_ values and lPAH concentration^66^. This is consistent with research on modern wildfires, the frequency of which is not only determined by the frequency of ignition agents (e.g. lightning strikes), but also by a number of variables including precipitation and net primary productivity^67^. Though global wildfire activity in the Holocene may correlate with temperature^68^, records of Holocene fire frequency in the Armenian Highlands co-varied with vegetation and aridity changes^69^.

At LKT1, the most robust evidence for fire (hPAHs) correlates with periods during which wildfires (lPAHs) were at their lowest frequency and occupation intensity was at its highest. Therefore, we can reject the hypothesis that fire use among MP hominins was predicated on its natural occurrence in the regional environment. We interpret the evidence as indicating the habitual use of fire by MP hominins during periods of low wildfire frequency. While these data do not preclude the interpretation that MP hominins were harvesting and maintaining wildfires, given that wildfire was still present on the landscape during all periods of occupation at the site, we conclude that the combined evidence demonstrate that MP hominins exhibited significant control over fire, and likely pyrotechnology.

These results suggest that pyrotechnology existed among MP hominins. However, its true antiquity has yet to be determined, and evidence for fire creation exists at 50 ka^13^ and evidence for its control dates to earlier than 300 ka^6,14^. If the contradictory evidence from MP sites in France is correct, these data suggest that MP fire use was regionally differentiated. This would suggest that the ability of hominins to control and perhaps create fire was either (1) a primitive behavioral trait that was lost in certain MP populations, or (2) a behavioral trait derived independently among MP populations. In either case, it follows that pyrotechnology was not limited to Upper Palaeolithic *Homo sapiens* and therefore unlikely to have played any role in the demise of MP hominins such as the Neanderthals. By applying the methods outlined here to Palaeolithic sites elsewhere in Eurasia and Stone Age sites in Africa, we will be able to determine what, if any, spatio-temporal patterns exist in pyrotechnology, and how such patterns might be equated with particular hominin species, environments, or climates.

## Methods

### Sample collection and extraction

We collected 18 sediment samples (~150 g) in the summer of 2014 from each sedimentary unit in Profile 4 at LKT1. We stored these samples at −20 °C, and they were lyophilized prior to lipid extraction. Lipids were extracted from the sediment via Soxhlet extraction in 300 mL of 2:1 dichloromethane:methanol for 48 hours. Following lipid extraction, we saponified the total lipid extract by heating the sample for 2 hours at 85 °C with 5 mL of 1 M KOH in methanol. We stopped the reaction by cooling to room temperature and adding 5 mL of 5% sodium chloride in water. We then extracted neutral compounds with a liquid/liquid extraction. *n*-alkanes and PAHs were separated from total liquid extract by passing samples through a column of activated silica gel (1.25 g) in baked Pasteur pipettes with 2 mL hexane (non-polar fraction), 4 mL dichloromethane (slightly polar fraction) and 4 mL methanol (polar fraction). *n*-alkanes were quantified through the analysis of the hexane fraction and PAHs were quantified through the analysis of the dichloromethane fraction. We did not identify PAHs in the hexane soluble fraction.

### Compound quantification

*n*-alkanes and PAHs were measured on a Trace Gas Chromatograph (GC) Ultra (Thermo-Scientific) fitted with a split–splitless (SSL) injector and flame ionization detector (FID). We quantified *n*-alkanes using a BP-5 column (30 m × 0.25 mm i.d., 0.25 μm film thickness) with He as the carrier (1.5 ml/min). Oven temperature was set at 50 °C for 1 min, ramped to 180 °C at 12 °C/min, then ramped to 320 °C at 6 °C/min and held for 4 min. We measured PAHs using a DB-5 column (60 m × 0.25 mm i.d., 0.25 μm film thickness) with He as the carrier (1.5 ml/min). Oven temperature was set at 70 °C and held for 4 minutes, ramped to 180 °C at a rate of 15 °C/min, ramped to 290 °C at 4 °C/min, then ramped to 310 °C at 5 °C/min and held for 19 minutes. Samples were analyzed in concert with a 16 component PAH standard (Restek SV Calibration Mix #5/610 PAH mix, Bellefonte PA, USA) at known concentrations ranging from 0.25 to 400 ng for calibration and quantification. Calibration mixes were also analyzed via Gas Chromatography-Mass Spectrometry (GC-MS) on an Agilent 6890 at the University of Connecticut with a relative standard error of 4%.

### Stable isotope analysis

The δD and δ^13^C values of individual *n*-alkanes were measured with a GC-Isolink coupled to a MAT 253 Isotope Ratio Mass Spectrometer (IRMS) (Thermo Scientific) with a BP-5 column (30 m × 0.25 mm i.d., 0.25 μm film thickness) at the University of Connecticut. Oven temperature and ramp was identical to the method used for *n*-alkane measurement. Isotopic standards (Mix A from A. Schimmelman) were analyzed every four samples across a range of concentrations to correct for size and scale effects. Standard deviations were 0.5‰ for δ^13^C (n = 7) and 4‰ for δD (n = 9). Stable isotopic composition is expressed in standard delta notation relative to VPDB and VSMOW.

## Supplementary information

Supplementary Information

## Data Availability

All data discussed in the paper is in this article and its Supplemental Information.
