# Supplementary material for: Geochemical Evidence for the Control of Fire by Middle Palaeolithic Hominins
Source: Sci Rep. 2019 Oct 25;9:15368. doi: 10.1038/s41598-019-51433-0 (PMC6814844; doi:10.1038/s41598-019-51433-0)
Supplement: Supplementary file 1 — Supplementary Information [file 41598_2019_51433_MOESM1_ESM.docx]

Geochemical Evidence for the Control of Fire by Middle Palaeolithic Hominins

Supplementary Information

Alex Brittingham*^1^, Michael T. Hren^2,3^, Gideon Hartman^1,4^, Keith N. Wilkinson^5^, Carolina Mallol^6,7,8^, Boris Gasparyan^9^, and Daniel S. Adler^1^

*Correspondence to alexander.brittingham@uconn.edu


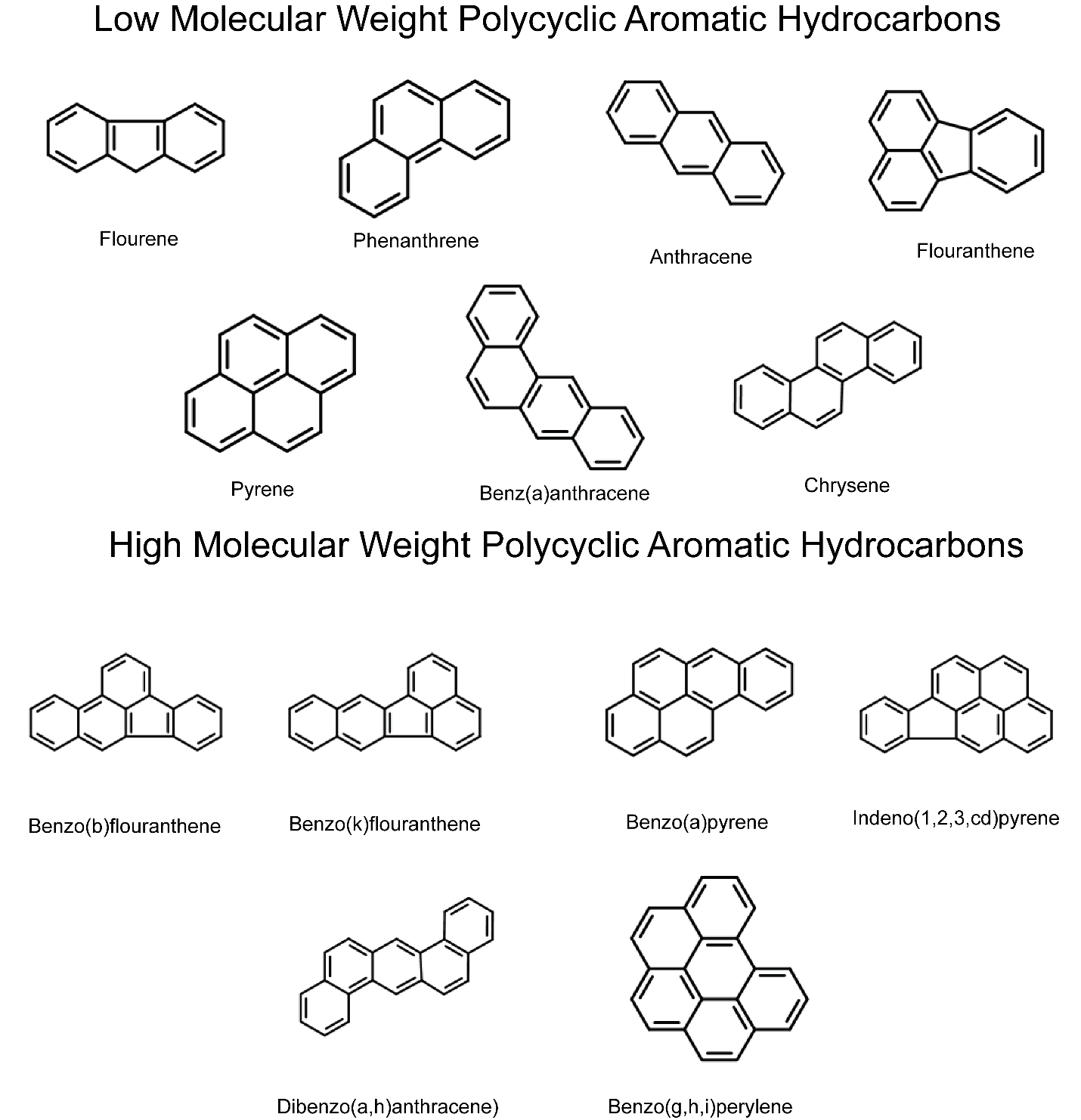


**Supplemental Figure S1:** Polycyclic aromatic hydrocarbons included in this study. Three- and four-ringed PAHs are considered low molecular weight, and five- and six-ringed PAHs are considered high molecular weight

| **Layer** |  | **ACL**^1^ |  | **OEP**^2^ |  | ***n*C_31_/*n*C_23_** |  | **Conc.^3^** |
| --- | --- | --- | --- | --- | --- | --- | --- | --- |
| **3** |  | 28.8 |  | 8.7 |  | 12.0 |  | 402.0 |
| **4.6** |  | 29.4 |  | 8.2 |  | 7.0 |  | 203.0 |
| **4.8** |  | 28.8 |  | 11.6 |  | 24.8 |  | 779.4 |
| **4.1** |  | 29.2 |  | 11.9 |  | 18.7 |  | 458.6 |
| **5.1** |  | 29.2 |  | 12.6 |  | 15.8 |  | 391.0 |
| **5.8** |  | 29.0 |  | 12.6 |  | 31.9 |  | 907.1 |
| **5.9** |  | 29.1 |  | 13.3 |  | 47.6 |  | 595.3 |
| **5.2** |  | 29.4 |  | 12.2 |  | 38.8 |  | 1083.1 |
| **6.3** |  | 29.5 |  | 12.1 |  | 52.7 |  | 1102.4 |
| **6.5** |  | 29.6 |  | 13.2 |  | 52.4 |  | 591.4 |
| **6.6** |  | 29.6 |  | 13.8 |  | 58.8 |  | 757.5 |
| **6.9** |  | 29.6 |  | 13.3 |  | 26.5 |  | 1156.7 |
| **6.8** |  | 29.8 |  | 14.4 |  | 33.0 |  | 1195.2 |
| **F** |  | 29.7 |  | 13.1 |  | 41.8 |  | 1010.0 |
| **7** |  | 29.3 |  | 12.7 |  | 16.9 |  | 1058.8 |
| **10.1** |  | 29.6 |  | 12.6 |  | 32.7 |  | 965.9 |
| **10.2** |  | 29.6 |  | 7.9 |  | 12.6 |  | 644.3 |
| **10.3** |  | 29.3 |  | 12.2 |  | 30.8 |  | 758.6 |

1: $OEP = \frac{C_{25}+C_{27}+C_{29}+C_{31}+C_{33}}{C_{24}+C_{26}+C_{28}+C_{30}+C_{32}}$

2: $ACL = \frac{23\times C_{23}+25\times C_{25}+{27\times C}_{27}+{29\times C}_{29}+{31\times C}_{31}+33\times C_{33}}{C_{23}+C_{25}+C_{27}+C_{29}+C_{31}+C_{33}}$

3: Sum of alkanes *n*C_23_ to *n*C_33_ (µg/g sediment extracted)

**Supplemental Table S2**: Average chain length and odd-over-even predominance for *n*-alkanes from each sedimentary unit at LKT1.


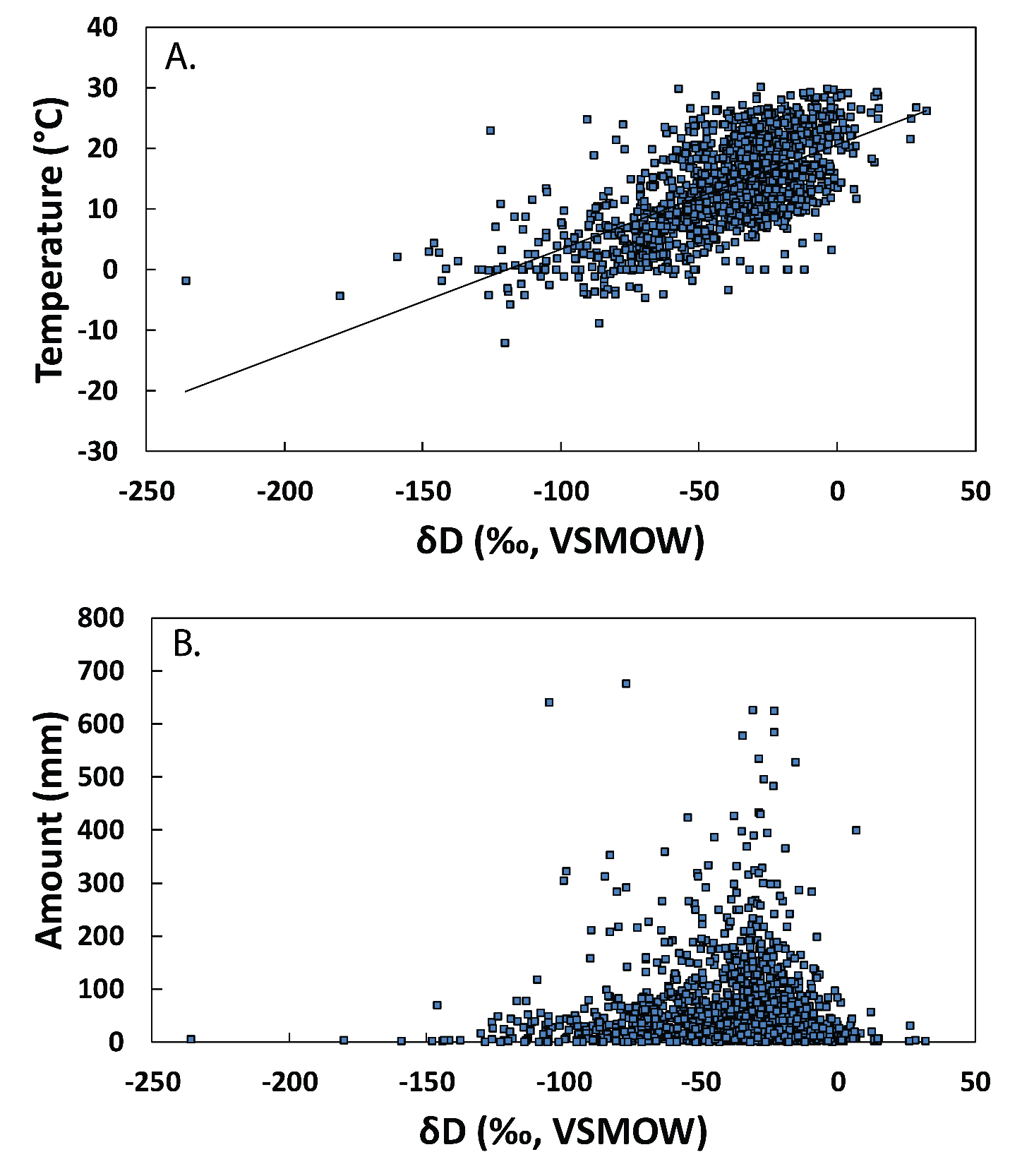


**Supplementary Figure S3**: δD values of monthly precipitation samples (n=1304) compared to temperature (A) and amount of precipitation (B) from the Global Network of Isotopes in Precipitation (GNIP) stations in Georgia and Turkey.

**Supplemental Discussion S4:**

**Lusakert Cave 1 (LKT1) Research History, Excavation Methods, Lithostratigraphy, Soil and Sediment Micromorphology**

**Research History**

Excavations at Lusakert caves 1 and 2 were first conducted between 1971–1981 by B. Yeritsyan of the Institute of Archaeology and Ethnography, Armenian Academy of Sciences, followed by an Armenian-French team in the early 1990s. Our joint Armenian-American-British team carried out a geoarchaeological assessment of the existing sections in 2007, and between 2008 and 2011 four excavation seasons were undertaken, followed by a study season in 2012. In 2008, efforts focused outside the cave where Yeritsyan, and later the Armenian-French team had worked and where eroded sections were still visible and easily accessible. We exposed new sections, clarified the original stratigraphy, collected a variety of chronometric and palaeoenvironmental samples, recovered several thousand lithic artifacts and bones, and began a geoarchaeological assessment of the site and the formation processes that influenced its development. No samples discussed in this paper were collected from the exterior portion of the site.

In 2009, we opened a 2x2 meter *sondage* within the cave and encountered stratified archaeological horizons rich in lithics, fauna, microfauna, and combustion structures. The cave opening is approximately 12 meters wide and is no more than 5 meters deep from the drip line. Over the next three field seasons we expanded and excavated to bedrock a 7m^2^ area, producing seven connected profiles that link the new deposits in the interior of the cave with those previously documented outside the cave. These excavations extended no more than 2 meters into the cave from the drip line. Profiles 3 and 4 represent the type sections for the interior deposits and it was from these that the vast majority of sediment, micromorphology, and chronometric samples derive, including all of those discussed in this paper.

Within the interior assemblage, the predominant flaking technique is Levallois and Kombewa (or Janus flakes), and resembles Middle Palaeolithic assemblages found elsewhere in the Southern Caucasus^1,2^. All sediments were water-screened and produced very high frequencies of fauna, microfauna, and small lithic debris, indicating all stages of reduction are represented. Although a concerted effort was not made to refit any of the artifacts, during analyses several refit groups were nonetheless identified within, but not between stratigraphic units during analyses. Additional information is available concerning the lithic assemblage and the sourcing of the obsidian artifacts^3,4^.

**Excavation Methods**

An excavation grid of 1x1m squares was established on the site prior to archaeological excavations. The position of all lithic artifacts larger than 2 cm, fauna, samples and features was recorded in three dimensions using two Leica TCR805 total stations linked to laptop PCs running EDMWIN, an archaeologically specific GIS program. The total station was also used to collect measurements along stratigraphic boundaries between units in the fresh vertical stratigraphic sections that were then imported into NewPlot, another GIS program for analysis of point-provenanced artifacts. Samples were collected from the cleaned stratigraphic sections for luminescence dating, and micromorphological study. Columns of samples were taken at 0.02 m intervals for FTIR measurement, and phytolith and palynology study, as continuous 0.02 m-thick blocks for tephrochronological study, and in 0.05 m-thick blocks for grain size analysis and magnetic susceptibility measurement. In addition, all excavated sediment was retained, with appropriate provenance, and wet sieved through a 1.6 mm mesh and dried. All artifacts and wet-sieved samples were subsequently transported to the project laboratory in Yerevan where they were washed, labeled, bagged, photographed, analyzed, weighed, and sorted, and then transported to the Institute of Archaeology and Ethnography, Armenian Academy of Science, Yerevan for curation and storage.

**Lithostratigraphy**

The lithostratigraphy of LKT1 was described during the 2008–2011 excavations, while samples were also collected for sedimentological, geochemical and micromorphological analyses. Nineteen lithological units (i.e. strata with distinct lithological and morphological characteristics that could be clearly discriminated from surrounding deposits) and sixteen sub-units (i.e. lithofacies variants) were identified. Only those units/sub-units from inside LKT1 that were sampled for *n*-alkanes and PAHs are described in the text below and then solely on the basis of their properties as observed in the field. Sub-units are differentiated from the parent unit by numbers following a full-stop.

**Unit 10** forms the basal archaeologically-relevant stratum in the cave. It unconformably overlies a mafic lava that has been correlated with 'Basalt 1'^5^ and which has been dated by ^40^Ar/^39^Ar to 197 ± 7 Ka. Unit 10 is divided into three sub-units on the basis of color differences (10.1–10.3), but which can collectively be characterized as olive gray (Munsell: 5 Y 4/2) moderately sorted medium sand with frequent platy sub-angular coarse sand-sized and gravel-sized mafic basalt clasts. It is likely that the unit formed in an alluvial channel and at a time when the Hrazdan river occupied the meander immediately east of the site.

**Unit 7** unconformably overlies Unit 10. Both Unit 7 and the overlying Unit 6 are possibly derived from accumulation of clasts and clay re-worked from Unit 10 and from an alluvial floodplain stratum on the exterior of the cave, Unit D2. Unit 7 is a dark brown (10 YR 3/3) poorly sorted sandy-clay layer. The sand fraction comprises sub-angular, platy fragments of mafic basalt. A high frequency of gravel-sized charcoal fragments and obsidian artefacts demonstrate human activity during the formation of this layer. Its upper contact with Unit 6 is diffuse.

**Unit 6** contains plentiful evidence for human activity and comprises eight sub-units that are differentiated on the basis of morphological properties and color (Units 6.1–6.6 and 6.8–6.9). Variance in the latter suggests that deposition involved a complex series of depositional events. The unit can broadly be categorized as a brown (10 YR 4/3) to dark grayish brown (10 YR 3/2) moderately sorted sandy-clay containing frequent obsidian artifacts. Internal contacts are both diffuse and sharp, while the upper boundary with Unit 5 is also diffuse.

**Unit 5** comprises nine sub-units (Units 5.1–5.9), but can be broadly categorized as a dark yellowish brown (10 YR 4/4) moderately sorted sandy clay with common gravel-sized mafic lava fragments, obsidian artifacts and charcoal fragments. As with Unit 6, the sub-units of Unit 5 are defined on the basis of grain size variation, gravel-sized inclusions and color. Unlike Unit 6, Unit 5 contains frequent vertical, calcareous rhizoconcretions as well as infilled small mammal burrows. The source material for Unit 5 (and of the overlying Units 4–2) is possibly floodplain alluvium that sits east of the cave (i.e. Units D1A–D1B), while secondary components are eroded fragments of the cave wall and the bi-products of human activity (obsidian artifacts and charcoal fragments). There are clay coatings on many of the clasts suggesting translocation, and these data together with the secondary carbonates suggests accumulation in a relatively warm environment.

**Unit 4** also consists of nine sub-units (Units 4.1–4.9) separated on the same basis as Unit 5. The stratum can be generalized as very dark grayish brown (10 YR 3/2) poorly sorted silt to medium sand and containing high frequencies of obsidian artifacts. The stratum contains moderate quantities of gravel-sized charcoal fragments suggesting that it incorporates the reworked residues of fire, while Units 4.7 and 4.9 are discrete areas of blackened and rubified sediment indicative of in situ combustion structures. As with Unit 5, Unit 4 contains several small mammal burrows indicating that localized mixing has occurred.

**Unit 3** is separated by a diffuse boundary from Unit 4, is not sub-divided and comprises dark brown (10 YR 3/3) poorly sorted silt to medium sand with common gravel-sized mafic basalt fragments and frequent obsidian artifacts. There is less evidence of burning in the form of charcoal than in the underlying Units 4–5.

**Soil and Sediment Micromorphology**

Moderate to frequent quantities of charcoal, which in Unit 5 (125–70 cm) are present as distinct fine horizontal layers, are indicative of anthropogenic combustion activity at the site. Microscopic burnt bone fragments are also ubiquitous throughout the stratified sequence. Furthermore, Unit 4 yielded an in-situ combustion structure as confirmed by its microstratigraphy, microscopic components and presence of residual microscopic calcareous ash (Extended Fig. 3).

The entire LKT1 sedimentary sequence shows weak ice micro-lensing, silty-clay cappings, vesicles, fissured charcoal and common redoximorphic features such as iron oxide nodules, mottling and staining. These features indicate recurrent, seasonal frost under moderately humid conditions.^6,7^At the same time, calcareous components and features, including calcitic hypocoatings, rhizoconcretions, plant ash and spherulite-rich coprolites are well preserved throughout the sequence, pointing to an alkaline sedimentary environment (Extended Table 4).

Microscopic-scale re-working of fine sedimentary particles resulting from the mentioned recurrent freeze-thaw and localized root and mesofauna bioturbation are the only agents of postdepositional disturbance identified micromorphologically. No evidence of leaching or any other vertical translocation process was observed.

| 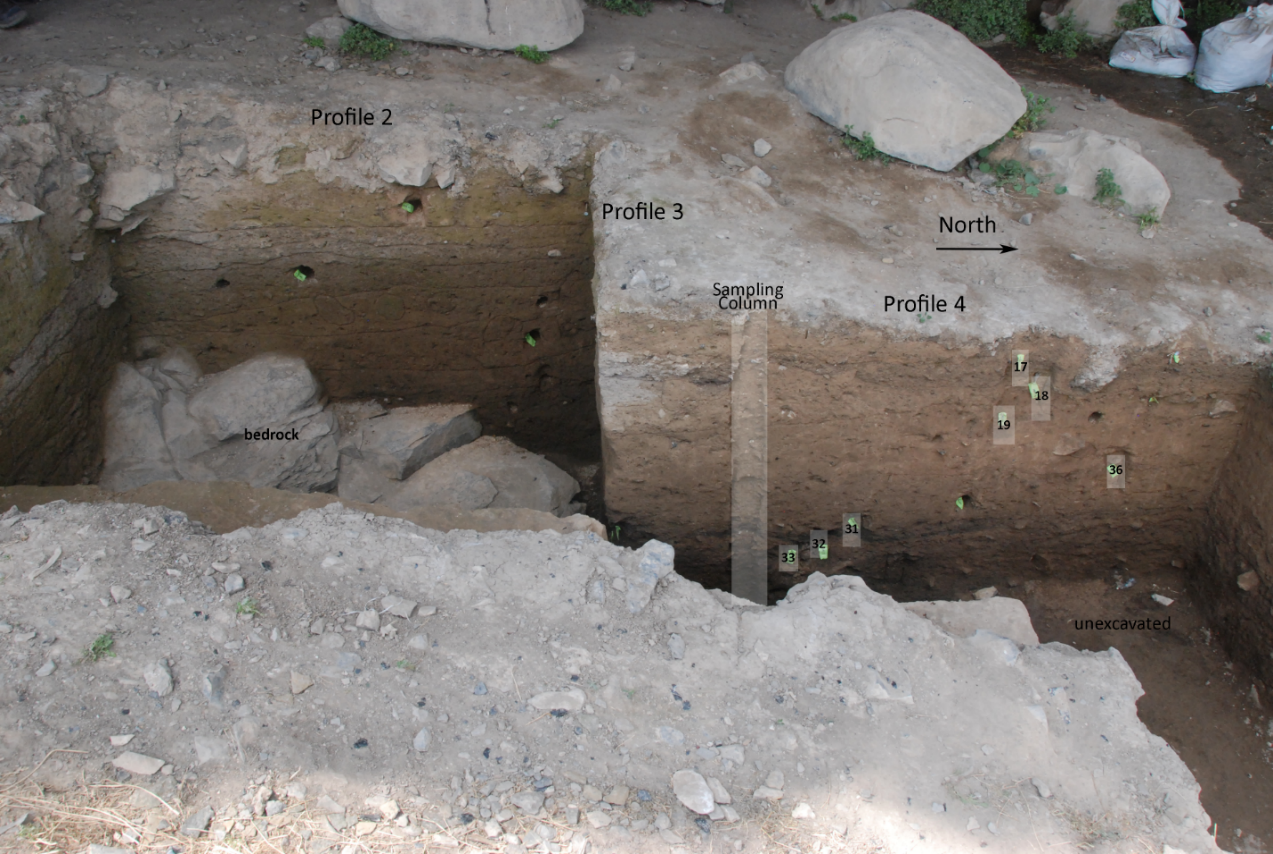  **A** |
| --- |
| 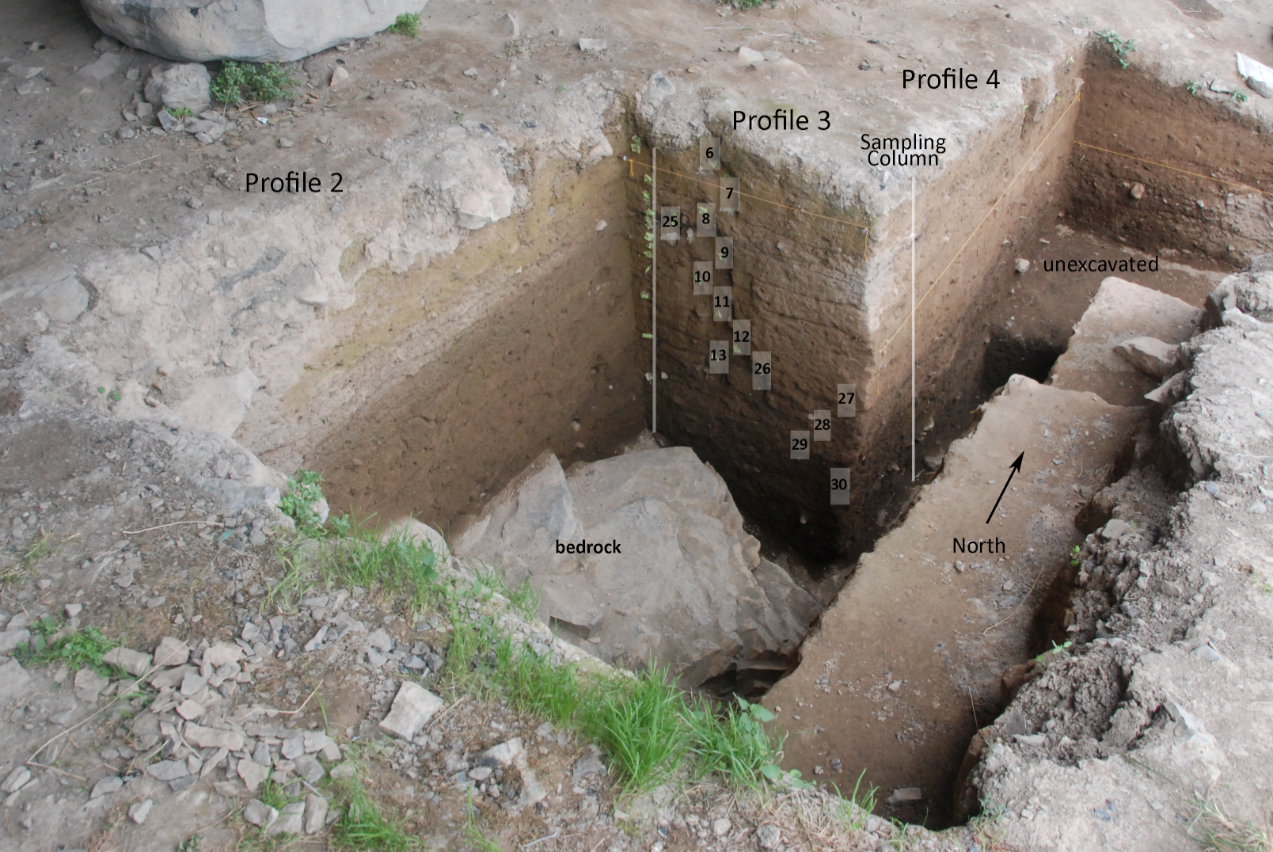  **B** |
| **Supplemental Figure S5**: **(A)** Interior of LKT1 (2011), with Profiles 2–4 and the locations of important micromorphology samples (numbered white boxes) and the column from which many of the samples discussed in this paper derive. **(B)** Interior of LKT1 (2011), with Profiles 2–4 and the locations of important micromorphology samples (numbered white boxes), in particular samples 8 and 25, and the column from which many of the samples discussed in this paper derive. |


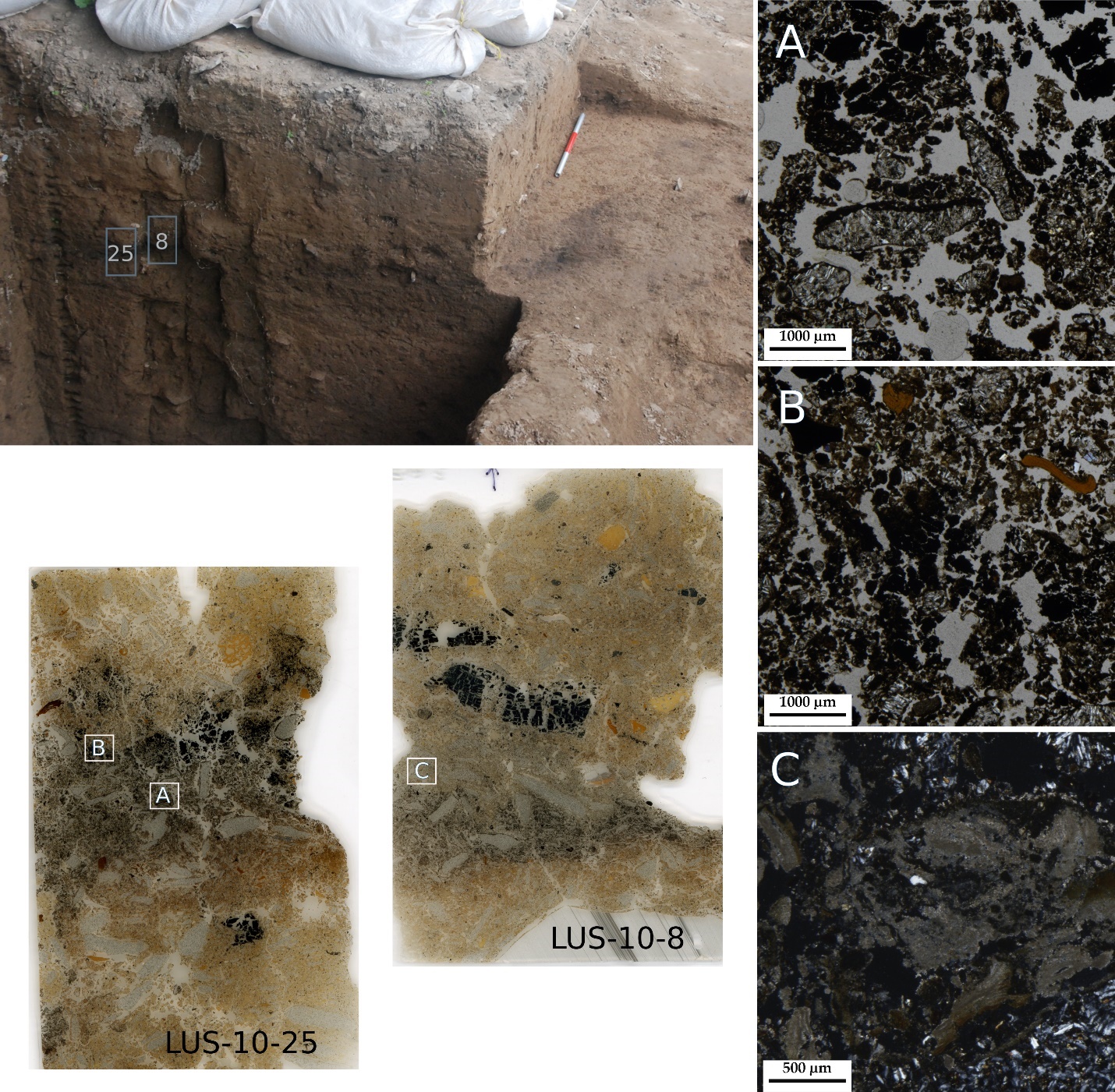


**Supplemental Figure S6**: Evidence of in situ anthropogenic fire at LKT1. Micromorphological samples 8 and 25 taken from Profile 3 (2009) show evidence of in situ anthropogenic combustion. As shown in the field photograph (2010, scale 30 cm), a large (>2 cm thick) black lens was observed toward the top of the section in Layer 4 (Layer 4.9 combustion feature). The thin section scans show a characteristic anthropogenic combustion structure stratigraphy (Mallol et. al., 2017) consisting of a black, 1–2 cm-thick sedimentary layer underlain by thinner reddish layer. Sample 25 also shows a 1 cm-thick light gray layer on top of the black one, and is capped by charcoal fragments. Both samples contain burnt bone fragments, which concentrate toward the top of the black and light-gray layers. Both combustion structures were post-depositionally cryoturbated, as indicated by a combination of platy microstructures, abundant fissures, silt-capped rock fragments and shattered state of the charcoal fragments, features common throughout the LKT1 sedimentary sequence. Despite of these recurrent disturbance processes, which possibly caused lateral stretching and minor internal reworking, the combustion structure remains in situ. A. Charred organic-rich sediment and two silt-capped bedrock fragments in the combustion structure's black layer, plane polarized light (PPL). B. Detail of the charred organic-rich sediment and two burnt bone fragments in the combustion structure's black layer, PPL. C. Residual calcitic wood ash in the light gray layer, cross polarized light (XPL).


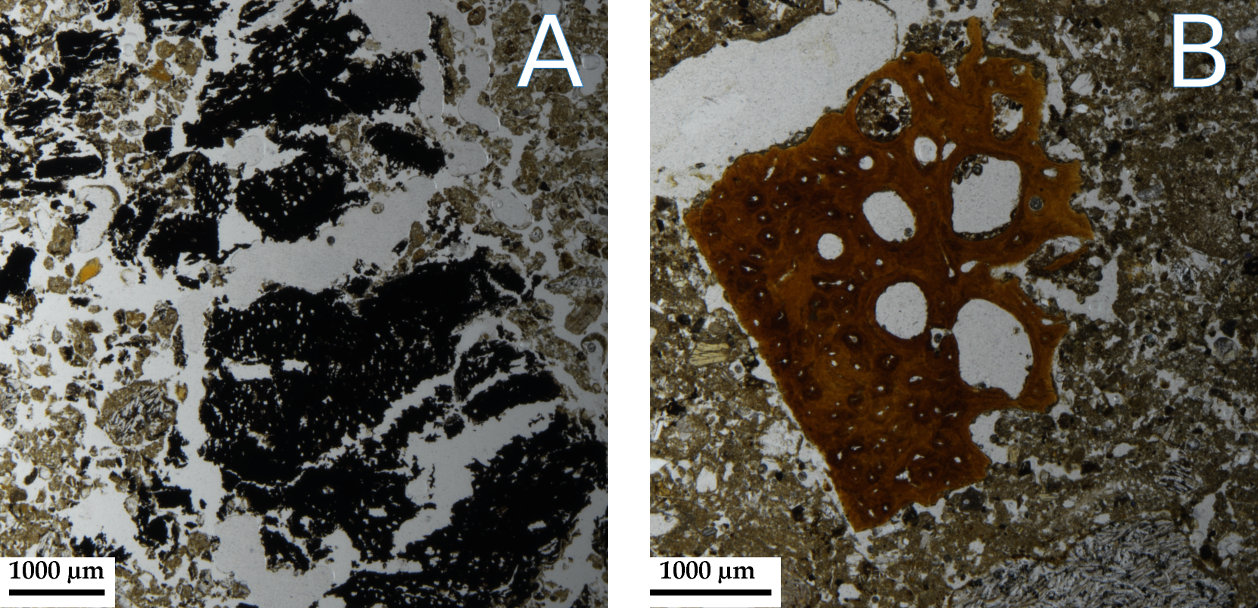


**Supplemental Figure S7**: Representative examples of microscopic charcoal (A) and burnt bone (B) fragments found throughout the LKT1 sedimentary sequence, PPL.

| **Layer** |  | **Unburnt**  **Bone** |  | **Burnt**  **Bone** |  | **Charcoal**  **Fragments** | **Obsidian**  **Artefacts** |  |  | **Spherulite-containing**  **Coprolites** |  | **Seasonal Freeze-Thaw*** | **Root Bioturbation†** |
| --- | --- | --- | --- | --- | --- | --- | --- | --- | --- | --- | --- | --- | --- |
| **2** |  | X |  |  |  |  |  |  |  | X |  | X | H,R |
| **3** |  |  |  |  |  |  |  |  |  | X |  | X | H,R |
| **4** |  | X |  | X |  | X | X |  |  | X |  | X | H,R |
| **5** |  | X |  | X |  | X | X |  |  | X |  | X | H |
| **6** |  | X |  | X |  | X | X |  |  | X |  | X | H |
| **10** |  | X |  | X |  | X | X |  |  |  |  | X | H |

*: As evidenced by presence of silty-clay cappings, subhorizontal micro-lensing and iron oxide nodules, mottles and staining.

†: As evidenced by root hypocoatings in channels (H) and rhizoconcretions (R).

**Supplemental Table S8**: Main micromorphological components and features observed in the LKT1 sedimentary sequence.

| **Layer** |  | **B(b)f*** |  | **B(k)f*** |  | **B(a)p*** |  | **[Ip,Da]*^a^** |  | **B(g,h,i)p*** |  | **hPAH** |
| --- | --- | --- | --- | --- | --- | --- | --- | --- | --- | --- | --- | --- |
| **3** |  | 15.2 |  | 16.3 |  | 3.9 |  | 5.1 |  | 3.6 |  | 44.1 |
| **4.6** |  | 8.5 |  | 14.4 |  | 20.9 |  | 5.9 |  | 2.1 |  | 51.8 |
| **4.8** |  | 14.3 |  | 11.8 |  | 10.1 |  | 4.5 |  | 0.7 |  | 41.4 |
| **4.1** |  | 12.1 |  | 14.5 |  | 16.3 |  | 6.1 |  | 1.9 |  | 51.0 |
| **5.1** |  | 32.5 |  | 51.5 |  | 78.7 |  | 13.6 |  | 1.6 |  | 178.0 |
| **5.8** |  | 26.8 |  | 74.1 |  | 29.0 |  | 14.0 |  | 2.0 |  | 146.0 |
| **5.9** |  | 46.2 |  | 1.9 |  | 130.0 |  | 15.9 |  | 2.0 |  | 195.9 |
| **5.2** |  | 27.1 |  | 55.2 |  | 330.3 |  | 11.4 |  | 2.1 |  | 426.1 |
| **6.3** |  | 24.0 |  | 35.4 |  | 115.5 |  | 11.8 |  | 1.5 |  | 188.2 |
| **6.5** |  | 8.9 |  | 43.0 |  | 97.0 |  | 18.6 |  | 3.6 |  | 171.0 |
| **6.6** |  | 22.8 |  | 42.0 |  | 142.6 |  | 19.0 |  | 1.2 |  | 227.5 |
| **6.9** |  | 0.4 |  | 76.1 |  | 31.6 |  | 10.7 |  | 1.6 |  | 120.4 |
| **6.8** |  | 7.4 |  | 59.5 |  | 5.9 |  | 14.4 |  | 2.8 |  | 90.0 |
| **F** |  | 4.9 |  | 10.8 |  | 0.9 |  | 4.9 |  | 2.4 |  | 23.8 |
| **7** |  | 4.6 |  | 12.9 |  | 1.0 |  | 5.5 |  | 2.1 |  | 26.1 |
| **10.1** |  | 8.9 |  | 6.6 |  | 2.9 |  | 3.7 |  | 2.6 |  | 24.7 |
| **10.2** |  | 8.3 |  | 10.8 |  | 3.4 |  | 5.1 |  | 1.8 |  | 29.3 |
| **10.3** |  | 9.2 |  | 9.9 |  | 3.1 |  | 4.4 |  | 1.0 |  | 27.5 |

**Supplementary Table S9**: Concentrations of lPAHs and hPAHs (ng/g sediment) from each sedimentary unit at LKT1.

| **Layer** |  | **Fle*** |  | **Phe*** |  | **Ant*** |  | **Fla*** |  | **Pyr*** |  | **B(a)a*** |  | **Chr*** |  | **lPAH** |
| --- | --- | --- | --- | --- | --- | --- | --- | --- | --- | --- | --- | --- | --- | --- | --- | --- |
| **3** |  | 30.8 |  | 43.2 |  | 47.3 |  | 3.7 |  | 27.7 |  | 4.2 |  | 2.5 |  | 159.3 |
| **4.6** |  | 50.6 |  | 27.5 |  | 32.1 |  | 6.3 |  | 52.3 |  | 1.4 |  | 1.4 |  | 171.6 |
| **4.8** |  | 10.9 |  | 72.3 |  | 84.7 |  | 10.0 |  | 13.0 |  | 3.3 |  | 1.9 |  | 196.2 |
| **4.1** |  | 3.4 |  | 60.9 |  | 87.1 |  | 7.7 |  | 10.4 |  | 1.9 |  | 2.5 |  | 174.0 |
| **5.1** |  | 3.4 |  | 19.0 |  | 35.7 |  | 6.8 |  | 15.2 |  | 18.6 |  | 2.4 |  | 101.0 |
| **5.8** |  | 16.2 |  | 64.9 |  | 83.5 |  | 4.4 |  | 10.1 |  | 1.5 |  | 1.5 |  | 182.1 |
| **5.9** |  | 4.7 |  | 35.4 |  | 48.4 |  | 4.5 |  | 10.8 |  | 2.9 |  | 2.8 |  | 109.4 |
| **5.2** |  | 8.2 |  | 10.6 |  | 21.9 |  | 3.3 |  | 8.4 |  | 1.0 |  | 1.1 |  | 54.6 |
| **6.3** |  | 1.7 |  | 12.6 |  | 15.2 |  | 5.1 |  | 12.4 |  | 4.8 |  | 2.1 |  | 54.0 |
| **6.5** |  | 4.0 |  | 23.7 |  | 33.2 |  | 9.3 |  | 14.3 |  | 7.7 |  | 3.4 |  | 95.6 |
| **6.6** |  | 2.3 |  | 12.1 |  | 34.7 |  | 11.7 |  | 10.7 |  | 2.1 |  | 2.2 |  | 75.8 |
| **6.9** |  | 1.4 |  | 11.2 |  | 20.7 |  | 5.3 |  | 7.3 |  | 10.1 |  | 1.9 |  | 58.0 |
| **6.8** |  | 11.1 |  | 10.1 |  | 26.2 |  | 3.4 |  | 6.5 |  | 19.7 |  | 2.6 |  | 79.6 |
| **F** |  | 2.2 |  | 14.4 |  | 22.0 |  | 2.2 |  | 7.3 |  | 12.0 |  | 2.0 |  | 62.0 |
| **7** |  | 2.0 |  | 14.1 |  | 17.9 |  | 4.4 |  | 19.9 |  | 4.3 |  | 2.3 |  | 65.0 |
| **10.1** |  | 2.9 |  | 6.5 |  | 7.3 |  | 3.1 |  | 12.7 |  | 4.8 |  | 1.6 |  | 39.0 |
| **10.2** |  | 1.1 |  | 22.2 |  | 25.9 |  | 2.9 |  | 7.5 |  | 0.7 |  | 1.0 |  | 61.3 |
| **10.3** |  | 1.2 |  | 17.3 |  | 21.2 |  | 1.4 |  | 3.6 |  | 1.8 |  | 1.7 |  | 48.3 |

*:Fle-flourene; Phe-phenanthrene; Ant-anthracene; Fla- Fluoranthene; Pyr-pyrene; B(a)a; Benz(a)anthracene, Chr-chrysene. *:B(b)f- benzo(b)fluoranthene; B(k)f-benzo(k)fluoranthene; B(a)p-benzo(a)pyrene; Ip- Indeno(1,2,3-cd)pyrene; Da- Dibenzo(a,h)anthracene; B(g,h,i)p- Benzo(ghi)perylene.

^a^-Ip and Da are summed due to coelution

**Supplementary Figure S10: PAH Normalization**

The measured concentration of PAHs preserved within a sedimentary deposit is affected by production, preservation, and accumulation within specific lithologic fractions. Changes in organic content, lithology, or processes associated with PAH synthesis (i.e. fire) may all play a role in shaping spatial or temporal trends in PAH abundance. There are a number of potential avenues for normalization of PAHs to minimize potential effects of lithologic heterogeneity through a stratigraphic sequence. The most common approach is to normalize PAH concentration is normalization relative to the dry weight of extracted sediment^8^. This approach yields a total PAH abundance per mass of soil, but does not account for variable clay or organic contents. Others suggest optimal normalization is relative to terrestrial organic content (TOC) or terrestrial biomarker abundance (e.g. alkanes, terpenoids, etc.)^9^. However, this approach assumes constant biomarker production through time. Numerous authors show strong differences in biomarker production with differing vegetation and/or ecosystem^10^, and others show that postdepositional degradation of molecular biomarkers may alter original molecular distributions^11,12^. We normalize PAH concentrations using both dry soil mass (A) and biomarker (n-alkane) abundance (B). Using either normalization method, PAH concentration is highest in Units 5 and 6, where artifact density is also highest. We suggest that at LKT1, due to minimal change in sediment lithology and the lack of clear relationship between PAH abundance and grain size distribution, that a simple normalization to dry mass is appropriate.


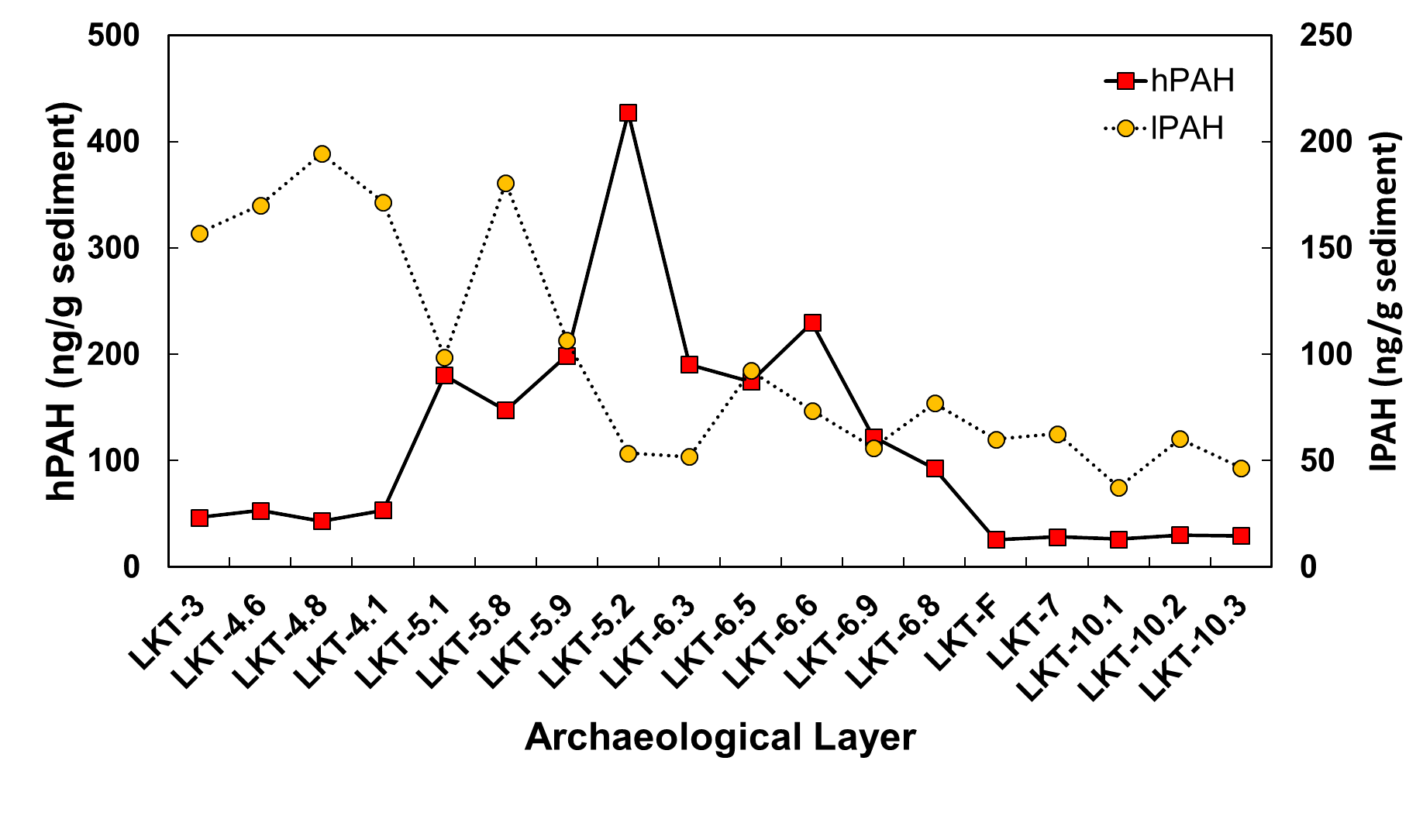


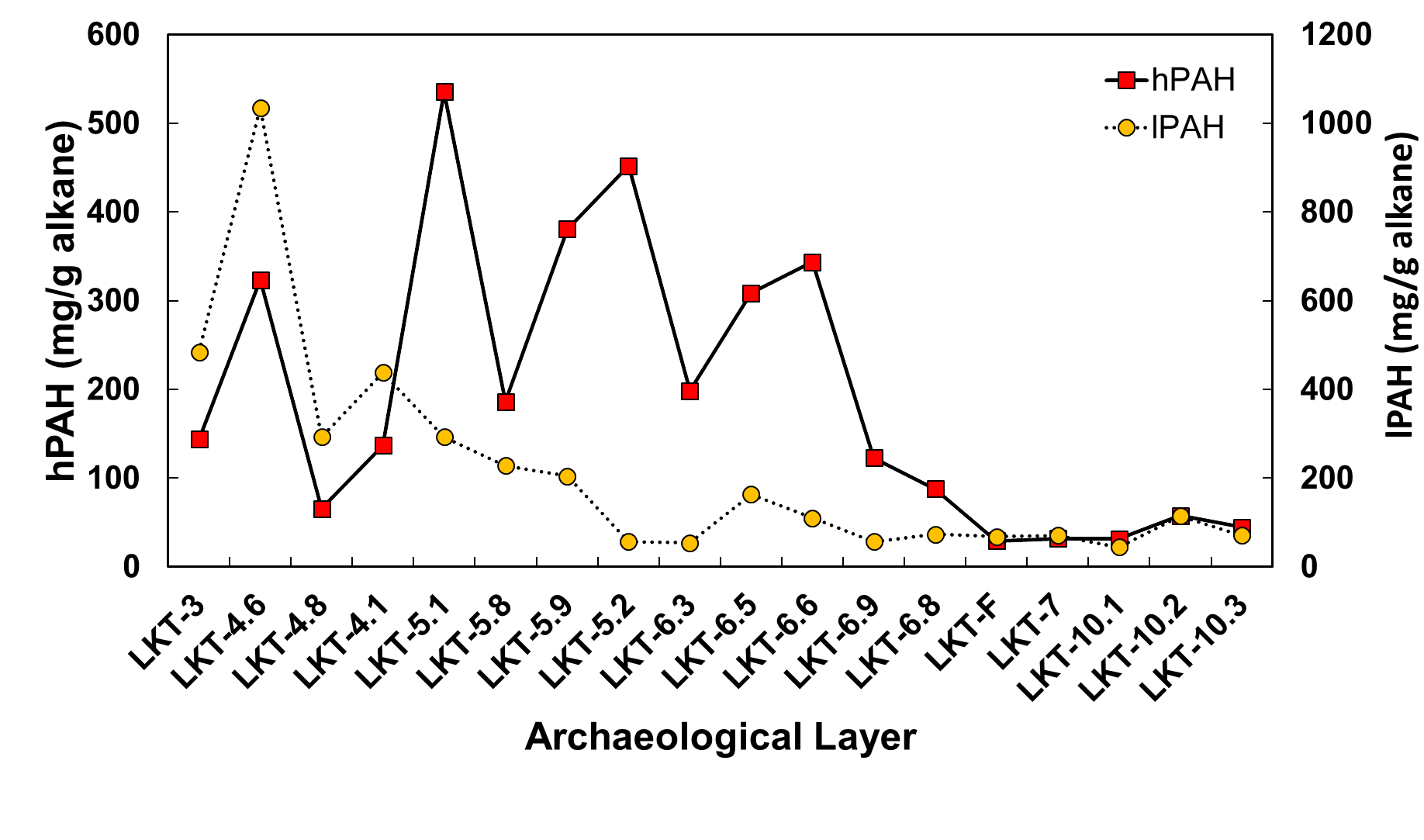


Supplementary Figure S10: lPAH (orange circles) and hPAH (red squares) concentration for each sedimentary unit of profile 4 at LKT1 when normalized to dry mass of sediment extracted (A) and long-chain (*n*-C_27_ to *n*-C_33_) *n*-alkane concentration (B)


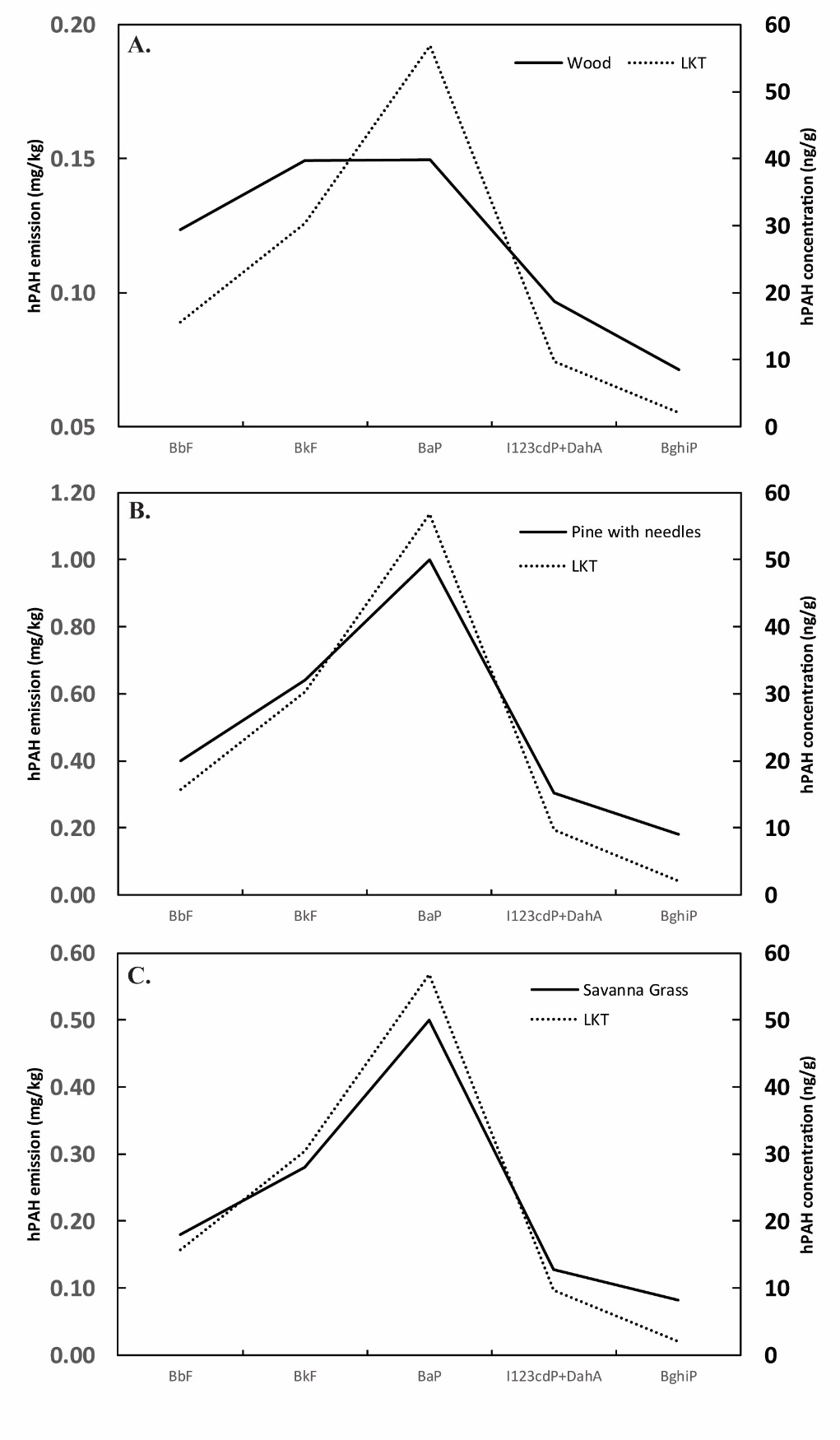
**Supplementary Information 11: hPAH Abundances in wood combustion**

**Supplemental Figure S11**: Mean hPAH concentration of sedimentary units at Lusakart Cave compared to modern studies on (A) wood combustion (Fine et al 2001, 2002, 2004a,b), (B) pine wood with needles (Iinuma et al 2007) and (C) savanna grass (Iinuma et al 2007).

SI References

1. Gasparyan, B. C. *et al.* The Middle Paleolithic Occupation of Armenia: Summarizing Old and New Data. in *Stone Age of Armenia: A Guide-book to the Stone Age Archaeology in the Republic of Armenia* (eds. Gasparyan, B. & Arimura, M.) 65–105 (Monograph of the JSPS-Bilateral Joint Research Project, Kanazawa University Press, 2014).

2. Adler, D. S. *et al.* The Hrazdan Gorge Palaeolithic Project, 2008-2009. in *Archaeology of Armenia in Regional Context, Proceedings of the International Conference dedicated to the 50th Anniversary of the Institute of Archaeology and Ethnography* (eds. Avetisyan, P. & Bobokhyan, A.) 22–38 (Gitutyun, 2012). doi:10.1017/CBO9781107415324.004

3. Frahm, E. *et al.* Lithic raw material units based on magnetic properties: A blind test with Armenian obsidian and application to the Middle Palaeolithic site of Lusakert Cave 1. *J. Archaeol. Sci.* **74,** 102–123 (2016).

4. Frahm, E. *et al.* Middle Palaeolithic toolstone procurement behaviors at Lusakert Cave 1, Hrazdan valley, Armenia. *J. Hum. Evol.* **91,** 73–92 (2016).

5. Adler, D. S. *et al.* Early Levallois technology and the Lower to Middle Paleolithic transition in the Southern Caucasus. *Science (80-. ).* **345,** 1609–1613 (2014).

6. Van Vliet-Lanoë, B. Traces de ségrégation de glace en lentilles associées aux sols et phénomènes périglaciaires fossiles. *Biul. Peryglac.* **26,** 41–54 (1976).

7. Van Vliet-Lanoë, B. Structures et microstructures associées à la formation de glace de segregation: leurs consequences. *Proc. 4th Int. Conf. Permafrost, Fairbanks, AK* 116–122 (1982).

8. Yunker, M. B. *et al.* PAHs in the Fraser River basin a critical appraisal of PAH ratio as indicators of PAH source and composition. *Org. Geochem.* **33,** 489–515 (2002).

9. Denis, E. H., Pedentchouk, N., Schouten, S., Pagani, M. & Freeman, K. H. Fire and ecosystem change in the Arctic across the Paleocene–Eocene Thermal Maximum. *Earth Planet. Sci. Lett.* **467,** 149–156 (2017).

10. Diefendorf, A. F., Freeman, K. H., Wing, S. L. & Graham, H. V. Production of n-alkyl lipids in living plants and implications for the geologic past. *Geochim. Cosmochim. Acta* **75,** 7472–7485 (2011).

11. Brittingham, A., Hren, M. & Hartman, G. Microbial alteration of the hydrogen and carbon isotopic composition of n-alkanes in sediments. *Org. Geochem.* **107,** 1–8 (2017).

12. Zech, M., Krause, T., Meszner, S. & Faust, D. Incorrect when uncorrected: Reconstructing vegetation history using n-alkane biomarkers in loess-paleosol sequences - A case study from the Saxonian loess region, Germany. *Quat. Int.* **296,** 108–116 (2013).
